# Supplementary material for: Effects of Different Light Spectra on Final Biomass Production and Nutritional Quality of Two Microgreens
Source: Plants (Basel). 2021 Jul 31;10(8):1584. doi: 10.3390/plants10081584 (PMC8399618; doi:10.3390/plants10081584)
Supplement: Supplementary file 1 [file plants-10-01584-s001.zip › plants-1309325-supplementary.pdf]

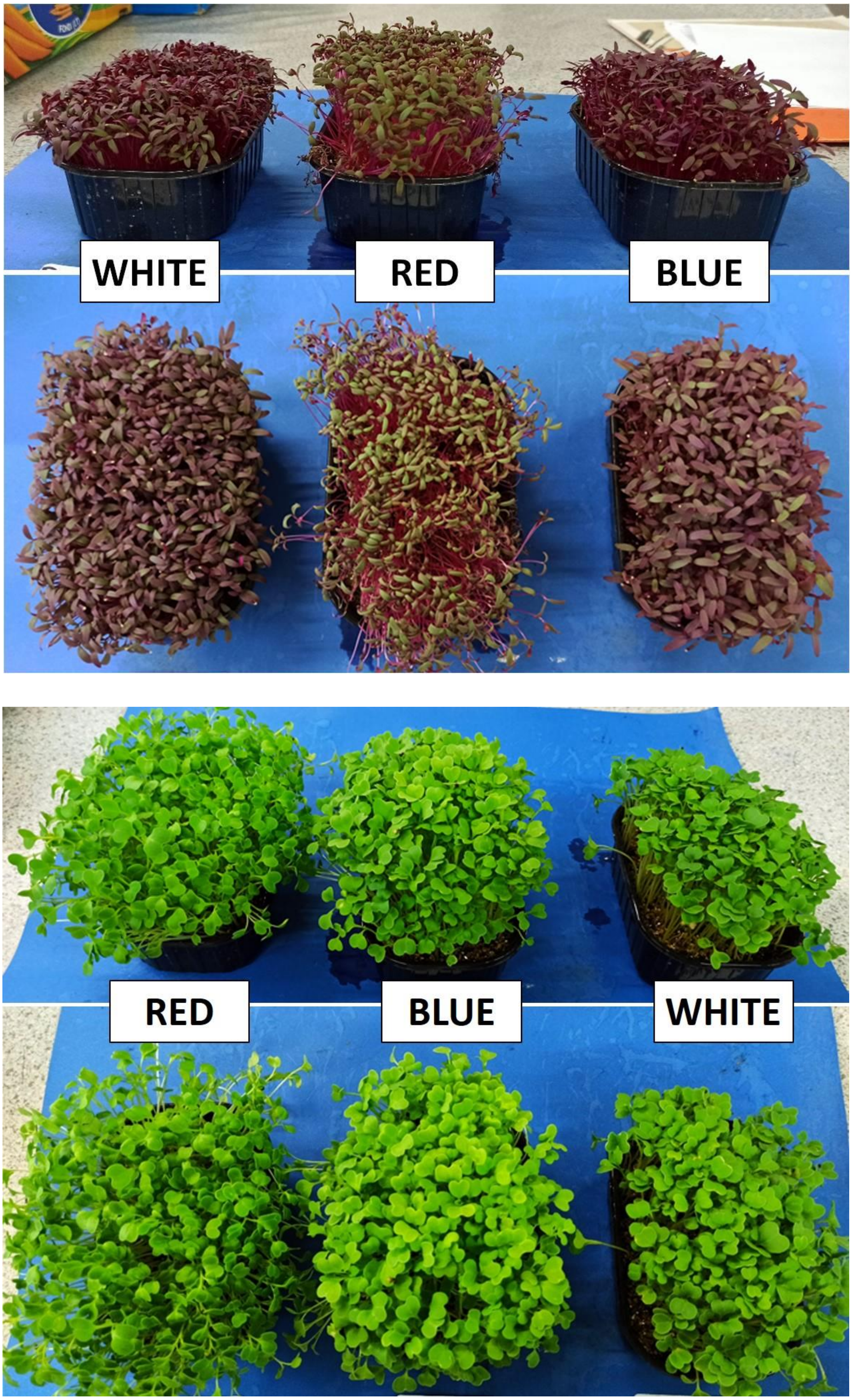

Figure S1. Amaranthus microgreens and Turnip greens microgreens 10 days from sowing at different LED light treatments.

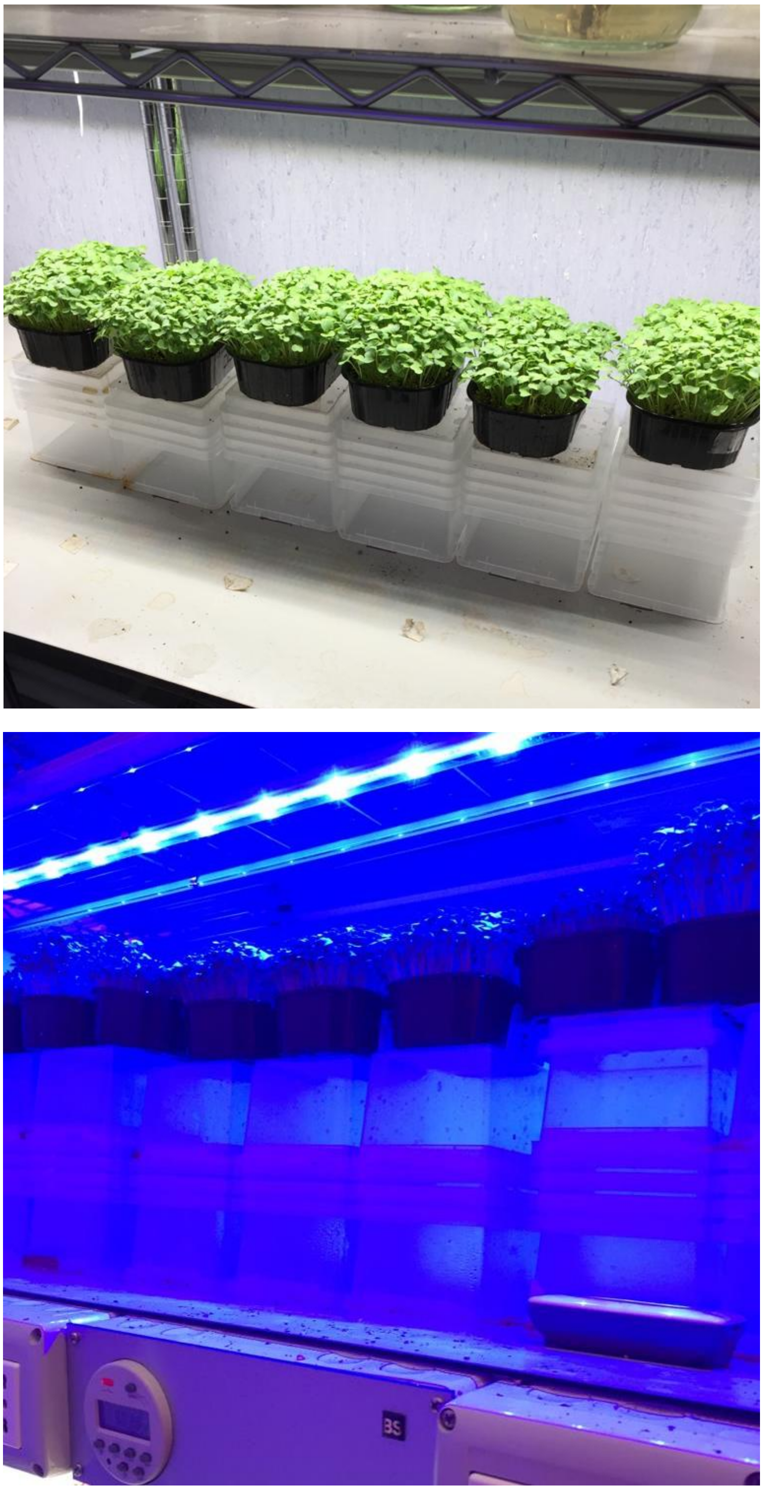

Figure S2. Microgreens in growth chamber at different LED light treatments (White and Blue).
